# Digital Immunophenotyping Predicts Disease Free and Overall Survival in Early Stage Melanoma Patients

**DOI:** 10.3390/cells10020422

**Published:** 2021-02-17

**Authors:** Francesco De Logu, Francesca Galli, Romina Nassini, Filippo Ugolini, Sara Simi, Mara Cossa, Clelia Miracco, Andrea Gianatti, Vincenzo De Giorgi, Eliana Rulli, Antonio Cossu, Daniela Massi, Mario Mandalà

**Affiliations:** 1 Section of Clinical Pharmacology and Oncology, Department of Health Sciences, University of Florence, 50100 Florence, Italy; francesco.delogu@unifi.it (F.D.L.); romina.nassini@unifi.it (R.N.); 2 Methodology for Clinical Research Laboratory, Oncology Department, Istituto di Ricerche Farmacologiche Mario Negri IRCCS, 20156 Milan, Italy; francesca.galli@marionegri.it (F.G.); eliana.rulli@marionegri.it (E.R.); 3 Section of Pathological Anatomy, Department of Health Sciences, University of Florence, 50100 Florence, Italy; filippo.ugolini@unifi.it (F.U.); sara.simi@unifi.it (S.S.); 4 Department of Pathology, Fondazione IRCCS Istituto Nazionale dei Tumori, 20133 Milan, Italy; mara.cossa@istitutotumori.mi.it; 5 Unit of Pathological Anatomy, Department of Medicine, Surgery, and Neurosciences, University of Siena, 53100 Siena, Italy; clelia.miracco@ao-siena.toscana.it; 6 Pathology Unit, Papa Giovanni XXIII Hospital, 24127 Bergamo, Italy; agianatti@asst-pg23.it; 7 Dermatology Unit, University of Florence, 50100 Florence, Italy; vincenzo.degiorgi@unifi.it; 8 Section of Pathology, Department of Medical, Surgical and Experimental Sciences, University of Sassari, 07100 Sassari, Italy; cossu@uniss.it; 9 Division of Pathological Anatomy, Papa Giovanni XXIII Hospital, 24127 Bergamo, Italy; 10 Unit of Medical Oncology, University of Perugia, 06123 Perugia, Italy

**Keywords:** melanoma, digital pathology, tumor infiltrating lymphocytes

## Abstract

Background: the prognostic significance of tumor infiltrating lymphocytes (TILs) in intermediate/thick primary cutaneous melanoma (PCM) remains controversial, partially because conventional evaluation is not reliable, due to inter-observer variability and diverse scoring methods. We aimed to assess the prognostic impact of the density and spatial distribution of immune cells in early stage intermediate/thick PCM. Materials and Methods: digital image acquisition and quantitative analysis of tissue immune biomarkers (CD3, CD4, CD8, CD68, PD-L1, CD163, FOX-P3, and PD-1) was carried out in a training cohort, which included patients with primary PCM ≥ 2 mm diagnosed, treated, and followed-up prospectively in three Italian centers. Results were validated in an independent Italian cohort. Results: in the training cohort, 100 Stage II–III melanoma patients were valuable. At multivariable analysis, a longer disease free survival (DFS) was statistically associated with higher levels of CD4^+^ intratumoral T-cells (aHR [100 cell/mm^2^ increase] 0.98, 95%CI 0.95–1.00, *p* = 0.041) and CD163^+^ inner peritumoral (aHR [high vs. low] 0.56, 95%CI 0.32–0.99, *p* = 0.047). A statistically significant longer DFS (aHR [high-high vs. low-low] 0.52, 95%CI 0.28–0.99, *p* = 0.047) and overall survival (OS) (aHR [high-high vs. low-low] 0.39, 95%CI 0.18–0.85, *p* = 0.018) was found in patients with a high density of both intratumoral CD8^+^ T-cells and CD68^+^ macrophages as compared to those with low density of both intratumoral CD8^+^ T-cells and CD68^+^ macrophages. Consistently, in the validation cohort, patients with high density of both intratumoral CD8^+^ and CD3^+^ T-cells were associated to a statistically better DFS (aHR[high-high vs. low-low] 0.24, 95%CI 0.10–0.56, *p* < 0.001) and those with high density of both intratumoral CD8^+^ and CD68^+^ were associated to a statistically longer OS (aHR[high-high vs. low-low] 0.28, 95%CI 0.09–0.86, *p* = 0.025). Conclusion: our findings suggest that a specific preexisting profile of T cells and macrophages distribution in melanomas may predict the risk of recurrence and death with potential implications for the stratification of stage II–III melanoma patients.

## 1. Introduction

The immune system plays an acknowledged role in melanoma development, progression and response to treatment [1,2,3]. Nevertheless, no biomarker has so far been translated so far into the clinic to define tumor immunity in individual patients. Instead, recognizing which patient has a “competent” immune system, prone to contribute to tumor control would help predicting patients who ultimately recur after surgery and those who do not. Melanoma is an archetype of immune surveillance theory, and it is recognized as one of the most immunogenic tumors [4]. 

The metastatic growth is a multistep process that involves interactions between the tumor and immune system [5]. As for other tumors, mortality is essentially linked to metastatic spread to sites that are distant from the primary tumor. Prognosticating outcome in early stage cutaneous melanoma is of paramount importance for several reasons: (i) to determine the need for further work-up investigations, (ii) to guide appropriate adjuvant treatment, (iii) to counsel patients, and (iv) to stratify those who enter clinical trials.

The conventional clinical and histopathological features that predict prognosis in primary cutaneous melanoma (PCM) include Breslow thickness (BT), ulceration, and sentinel lymph node (SN) status [6]. The presence of tumor infiltrating lymphocytes (TILs) in melanoma has been associated with a favorable prognosis in some studies [7,8,9,10,11,12,13] and implying a more effective host immunologic response to the tumor. However, other studies have failed to confirm such correlation [14,15], and the prognostic significance of TILs in thin vs. intermediate/thick PCM remains controversial.

TILs are a heterogeneous group of immune cells in the context of the tumor microenvironment mainly comprising of T-lymphocytes, and their assessment is subject to inter-observer variability, which can limit their applicability in routine use [15]. Among the immune cells that re recruited in the tumor microenvironment, macrophages are particularly abundant. Clinical and preclinical studies suggest that macrophages generally play a pro-tumoral role, by stimulating angiogenesis, enhancing tumor cell invasion, motility, and intravasation [16]. Spatial information and making inferences about the interactions of different immune cells, including macrophages with immunogenic and immunosuppressive functions, in the tumor tissue could represent a better way to investigate this complex scenario [17].

In order to assess the prognostic impact of the density and spatial distribution of immune cells in early stage intermediate/thick PCM, we herein evaluated TILs and macrophages by immunohistochemistry, digital image acquisition, and quantitative analysis to identify essential tissue immune biomarkers that are able to capture the immune contexture of the tumor microenvironment that could independently predict DFS and OS. 

## 2. Materials and Methods

### 2.1. Patients Characteristics

The cohort of the training set (*n* = 100) included patients with stage II–III intermediate/thick PCM ≥ 2 mm diagnosed, treated, and followed-up prospectively in four Italian centres (Istituto Nazionale Tumori, Milan; Dermatology Section, University of Florence, Florence, University of Sassari, Sassari and University Hospital of Siena, Siena, Italy) from 2000 to 2015. The clinical and histopathological parameters that were extracted from the database included: gender, date of birth, date of diagnosis of PCM, date of SN biopsy, BT, ulceration, SN status, surgical procedures, TILs, and follow-up, including the date of relapse and death. Haematoxylin and eosin slides were reviewed, and the histopathological features were re-assessed by two dedicated dermatopathologists (DM, MC). The tumor stage was assessed according to the American Joint Committee on Cancer (AJCC) TNM (Tumor, Node, Metastasis) [18]. The tumors were re-evaluated for lymphocytic infiltration in the vertical growth phase (VGP), and classified as brisk, non-brisk, and absent according to criteria that were formulated by Clark et al. [19]. Lymphocytes had to surround and disrupt tumor cells in the VGP to be defined as TILs. These lymphocytes were termed “brisk” if they infiltrated the entire invasive component diffusely or across the base of the VGP. TILs were “absent” if no lymphocytes were present or if they were present, but did not infiltrate the tumor. When lymphocytes only infiltrated the melanoma focally with one or scattered foci, the term “non-brisk” was used. 

The patients included in the validation cohort (*n* = 74) were treated at the Papa Giovanni XXIII Cancer Center Hospital, Bergamo, Italy. Similarly, to the training cohort, patients with BT ≥ 2 mm and with available tissue samples were included. Information on demographics as well as data on DFS and OS were retrieved for each patient. Data on treatment and survival were prospectively collected in the context of Melanoro project, as previously reported [14].

### 2.2. Tissue Samples

Formalin fixed paraffin-embedded (FFPE) tissue sections, 4 µm in thickness, were stained with haematoxylin and eosin and reviewed to confirm the histopathological diagnosis and assess tissue quality control.

### 2.3. Ethical Committee

Approval to conduct the study was obtained from the local Ethics Committees of the participating Centers. Specifically, the use of FFPE sections of human samples was approved by the Local Ethics Committee (13676_bio, protocol Id.21073) according to the Helsinki Declaration and informed consent was obtained.

### 2.4. Immunohistochemistry

Tumor immune infiltrate characterization was performed by evaluating eight immune cell markers (CD3, CD4, CD8, FOX-P3, PD-L1, PD-1, CD68, and CD163) via immunohistochemistry on representative FFPE whole tumor sections 3-μm thick of PCM. The sections were deparaffinized in EZ prep (950–102; Ventana), and antigen retrieval was achieved by incubation with cell-conditioning solution 1 (950–124; Ventana), a Tris ethylenediaminetetraacetic acid-based buffer (pH 8.2), for 32 min. at 100 °C or with Dako PT-link, EnVision™ FLEX Target Retrieval Solution, Low pH. Sections were incubated with the following primary antibodies: anti-CD3 (#790–4341, rabbit monoclonal, clone 2GV6 ready to use, Ventana Medical System, Tucson, AZ, USA), anti-CD4 (#790–4423, rabbit monoclonal, clone SP35, ready to use, Ventana Medical System, Tucson, AZ, USA), anti-CD8 (#790-4460, rabbit monoclonal, clone SP57, ready to use, Ventana Medical System, Tucson, AZ, USA), and anti-FOX-P3 (#b20034, mouse monoclonal, clone 236A/E7, 1:60. Abcam, Cambridge, UK). The signal was developed with the UltraMap Red anti-Mouse or anti-Rabbit Detection Kit (Ventana Medical Systems, Tucson, AZ, USA) in an automated Immunostainer (Ventana Discovery XT, Ventana Medical Systems, Tucson, AZ, USA). In addition, the sections were incubated with the following primary antibodies: anti-PD-L1 (clone 22c3 Dako Agilent, dilution 1:25), anti-PD1 (clone NAT105 Biocare; dilution 1:50), anti-CD68 (clone KP1, Dako Agilent, dilution 1:3000), and anti-CD163 (clone 10D6, Novus Biological, dilution 1:200). The signal was developed with EnVision™ FLEX+, Dako, Agilent) in an automated Immunostainer (Dako Autostainer Link 48). The sections were counterstained with hematoxylin. Appropriate positive controls were used throughout. 

Immunohistochemical scoring was performed in a blinded fashion by experienced melanoma pathologists (DM, MC). Stained sections were initially assessed at low magnification in order to select the areas with highest density of positive immune cells at peritumoral and intratumoral location. The assessment of immune cells score density was compared with evaluation that was obtained by image analysis. An evaluation of PD-L1 was performed in both immune cells and tumor cells, as previously described [20].

### 2.5. Image Analysis 

Stained tissue sections were digitally scanned at ×400 magnification with Aperio AT2 or Aperio ScanscopeXT platform (Leica Biosystems, Wetzlar, Germany) into whole slide digital images (WSI). Each SVS format file was imported into HALO Link^®^ (Indica Labs, Albuquerque, NM) image management system. Two expert pathologists (DM, MC) drew the image annotations of the whole surface and margins of PCM. The whole tumor area was defined as the area containing invasive tumor, including the invasive tumor borders, according to ITWG recommendations. 

**Figure 1:**
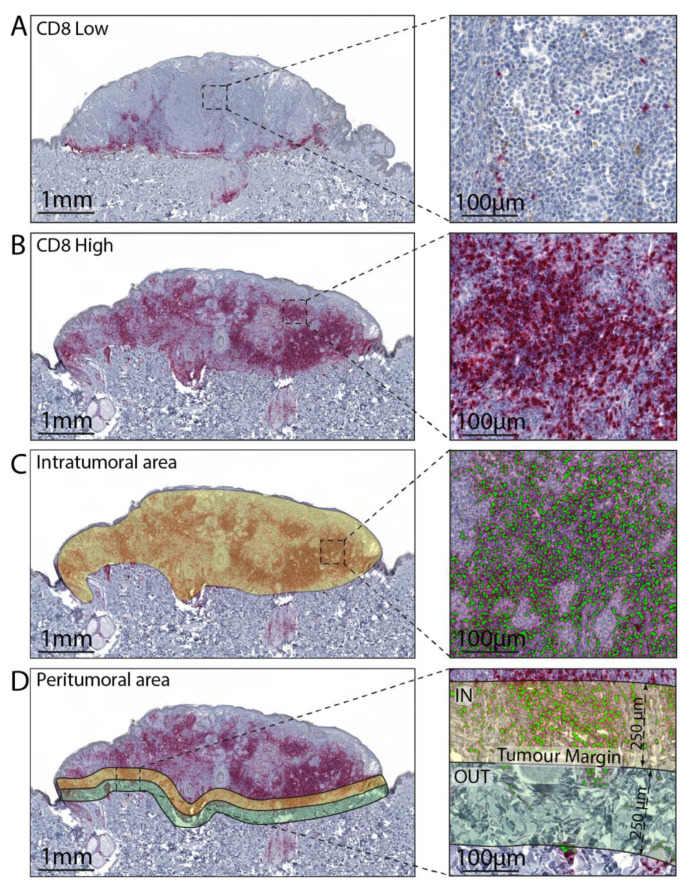
Representative images of melanoma tissue with low (**A**) and high (**B**) CD8 positive cells. (**C**) Representative annotation mask for intratumoral area and CD8 cells software recognition. (**D**) Representative annotation mask for inner (yellow, IN) and outer (green, OUT) portion of the peritumoral area and CD8 cells software recognition.

For all WSI, three different annotation layers were created, one for the intratumoral area and two, respectively, for the inner peritumoral area and outer peritumoral area, starting from the tumor border with a thickness of 250 µm (Figure 1). The detection of immune-stained positive cells, in the three different layers, was performed using HALO^®^ Multiplex IHC analysis software version v3.1.1076.308 (Indica Labs, Albuquerque, NM), based on cytonuclear features, such as stain intensity, size, and roundness for CD3, CD4, CD8, FOX-P3 CD68, CD163, PD-1, and PD-L1. The software automatically excludes tissue gaps from analysis and the settings were set-up to include the full range of staining intensity (from weak to strong). The data were expressed as cellular density (i.e., the number of positive cells divided by the mm^2^ of the annotation layer area).

### 2.6. Statistical Analysis

The aim of this study was to evaluate the prognostic value of intratumoral and peritumoral immune cell density on DFS and OS in intermediate/thick PCM. DFS was defined as the time between diagnosis and disease relapse or death from any cause. OS was defined as the time between diagnosis and death from any cause. Patients who had not relapsed/died or died were censored at the date of the last follow-up visit. Each immune cell biomarker was evaluated as a continuous variable and then categorized as low or high according to its median. 

Continuous variables were described using mean and standard deviation (SD), the median with the first and third quartiles (Q1–Q3; interquartile range, IQR), and minimum and maximum values, whereas the categorical variables were described using frequencies and percentages. Chi-square and Wilcoxon tests were performed in order to compare the distributions of categorical and continuous variable, respectively. The association between the immune cell biomarkers was assessed by means of the Spearman correlation index. A MANOVA analysis was performed for each immune cell biomarker to investigate the difference between intratumoral, inner and outer peritumoral area in terms of cells density. DFS and OS was evaluated using the univariable and multivariable Cox proportional hazard models. 

Multivariable models were adjusted for the BT, the ulceration, and stage. The results of the analyses were expressed as hazard ratios (HRs), adjusted HRs (aHRs), and 95% confidence intervals (95%CIs). The median DFS and OS were estimated with the Kaplan–Meier (KM) method. The same analysis carried out for the training cohort was adopted for the validation cohort using, as biomarker cut-offs, those calculated in the training cohort. Statistical significance was set at *p* < 0.05 for a bilateral test. Analysis was carried out using the SAS (Statistical Analysis System, SAS Institute, Version 9.4) software.

## 3. Results

### 3.1. Training Cohort

Overall, 100 stage II–III melanoma patients with BT ≥ 2 mm were included in the training cohort. Table 1 summarizes the demographical and clinical characteristics, whereas Table 2 provides the distribution of the biomarkers. The mean age of the patients was 63.2 years (SD 16.1), and 62 patients (62.0%) were male. Tables S1 and S2 report the association between the density of immune cells and their correlation with stage, BT, and ulceration. The median follow-up was 83.9 months (IQR 64.5–111.6). Overall, 46 patients (46.0%) relapsed, 52 patients (52.0%) died, and 52 patients (52.0%) relapsed or/and died (i.e., DFS events). The median DFS and OS were 38.4 months (IQR 12.2–129.7) and 85.7 months (IQR 38.0–162.2), respectively.

When comparing the three different regions (Table S3), we observed some striking spatial differences. A statistically significant higher density was found in the inner peritumoral area for CD3^+^ (*p* = 0.001), CD4^+^ (*p* < 0.001), and CD8^+^ (*p* = 0.002) as compared to the intratumoral area and for CD68^+^ (*p* = 0.035) and CD163^+^ (*p* = 0.003) compared to the outer peritumoral area (Figure S1). A longer DFS was statistically associated with higher levels of CD4^+^ intratumoral T-cells (aHR [100 cell/mm^2^ increase] 0.98, 95%CI 0.95–1.00, *p* = 0.041) and CD163^+^ inner peritumoral (aHR[high vs. low] 0.56, 95%CI 0.32–0.99, *p* = 0.047) (Table 3). A statistically positive impact on OS was found for higher levels of CD3^+^ outer peritumoral T-cells (aHR[100 cell/mm^2^ increase] 0.98, 95%CI 0.95–1.00, *p* = 0.044) and CD68^+^ intratumoral macrophages (aHR[100 cell/mm^2^ increase] 0.52, 95%CI 0.29–0.95, *p* = 0.033) (Table 3).

**Figure 2:**
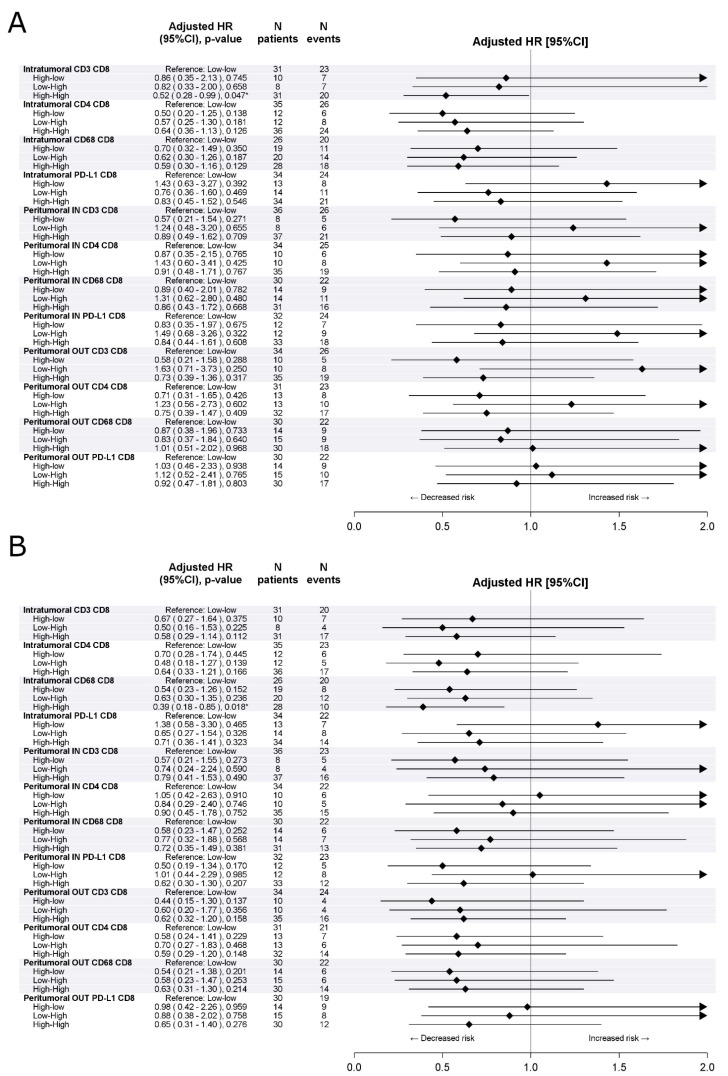
Effect of combination with CD8+ density on disease free survival (**A**) and overall survival (**B**) in the training cohort. Multivariable Cox proportional hazard models. * indicates significant *p*-value at 0.05 level.

Figure 2 and Figure S2 summarize the analysis of the prognostic impact of the combination of the density and distribution of CD8^+^ T-cells with the density and distribution of other immune cells in the tumor microenvironment. A statistically significant longer DFS (aHR[high-high vs. low-low] 0.52, 95%CI 0.28–0.99, *p* = 0.047) and OS (aHR[high-high vs. low-low] 0.39, 95%CI 0.18–0.85, *p* = 0.018) was found in patients with a high density of both intratumoral CD8+ T-cells and CD68^+^ macrophages as compared to those with a low density of both intratumoral CD8+ T-cells and CD68^+^ macrophages.

### 3.2. Validation Cohort

Overall, 74 stage melanoma patients with BT ≥2 mm were included in the validation cohort. Data on CD3^+^, CD4^+^, CD8^+^, and CD68^+^ image analysis were available. Table 1 and Table 2 show a comparison between the training cohort and the validation cohort in terms of demographical and clinical characteristics and distribution of immune cells. Tables S4 and S5 show the correlation between the density and spatial distribution of immune cells as well as their correlation with BT in the validation cohort. The median follow-up was 161.6 months (IQR 126.7–201.0). Overall, 39 patients (52.7%) relapsed, 37 patients (50.0%) died, and 41 patients (55.4%) relapsed or/and died (i.e., DFS events). The median DFS and OS were 88.3 months (IQR 14.7–301.1) and 140.8 months (IQR 36.1–301.1), respectively.

In line with data obtained in the training cohort, a statistically significant higher density was found in the inner peritumoral area when compared to the intratumoral density for CD3^+^ (*p* = 0.0101) and CD8^+^ (*p* = 0.0473) but not for CD4^+^. Moreover, for all the markers a statistically significant lower density was detected in the outer peritumoral area (Table S3).

At multivariable analysis (Table S6), a longer DFS was statistically associated with higher CD3^+^ intratumoral density (aHR[100 cell/mm^2^ increase] 0.95, 95%CI 0.91–0.99, *p* = 0.027; aHR[high vs. low] 0.38, 95%CI 0.19–0.78, *p* = 0.008) and higher CD3+ inner peritumoral density (aHR[100 cell/mm^2^ increase] 0.97, 95%CI 0.93–1.00, *p* = 0.030). Moreover, higher intratumoral CD8^+^ density correlated with both DFS (aHR[100 cell/mm^2^ increase] 0.94, 95%CI 0.88–0.99, *p* = 0.030; aHR[high vs. low] 0.23, 95%CI 0.10–0.50, *p* < 0.001) and OS (aHR[high vs. low] 0.30, 95%CI 0.13–0.70, *p* = 0.005). Lastly, a beneficial impact on DFS (aHR[100 cell/mm^2^ increase] 0.93, 95%CI 0.88–0.98, *p* = 0.006; aHR[high vs. low] 0.21, 95%CI 0.08–0.52, *p* < 0.001) and OS (aHR[100 cell/mm^2^ increase] 0.93, 95%CI 0.88–0.99, *p* = 0.015; aHR[high vs. low] 0.23, 95%CI 0.08–0.61, *p* = 0.003) was found in patients with high CD8^+^ density in the inner peritumoral environment.

**Figure 3:**
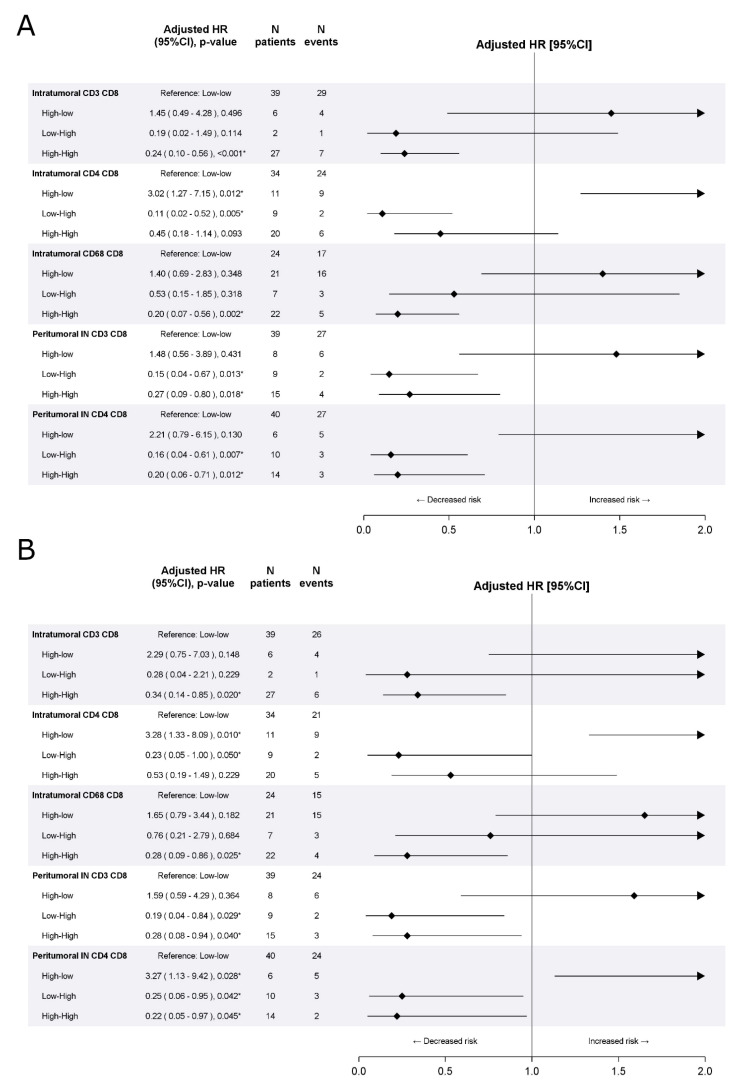
Effect of combination with CD8^+^ density on disease free survival (**A**) and overall survival (**B**) in the validation cohort. Multivariable Cox proportional hazard models.* indicates significant *p*-value at 0.05 level.

Figure 3 summarizes the impact of the combination of the density and spatial distribution of CD8^+^ T-cells with other immune cells on DFS and OS. Consistent with the training cohort, in the validation cohort, patients with high density of both intratumoral CD8+ and CD3^+^ T-cells had a statistically better DFS (aHR[high-high vs. low-low] 0.24, 95%CI 0.10–0.56, *p* < 0.001) and those with high density of both intratumoral CD8^+^ and CD68^+^ had a statistically better OS (aHR[high-high vs. low-low] 0.28, 95%CI 0.09–0.86, *p* = 0.025).

## 4. Discussion

The main result of our study is that the density and spatial distribution of CD8^+^ T-cells and macrophages in the microenvironment predict DFS and OS in clinical stage II–III intermediate/thick PCM patients. The AJCC staging system acknowledges BT, ulceration, and SN status as the most reliable prognostic factors and, in daily practice, the AJCC classification has a considerable and direct impact on cancer patients’ care. This is particularly true in the era of effective melanoma adjuvant therapies [21,22,23]. However, outcome prediction of the traditional staging system assumes that melanoma progression is a melanoma cell-autonomous process, and it does not consider the effects of the host immune response.

The interplay between melanoma and immune cells is a major determinant in melanoma progression and TILs are emerging as a powerful prognostic marker and therapeutic target in oncology [24,25]. Nevertheless, conventional evaluation of TILs is affected by interobserver variability and diverse scoring methods have been proposed [15,26]. Furthermore, TME includes a heterogeneous population, including not only T-lymphocytes, but also macrophages, and, to a lesser extent, B lymphocytes and natural killer cells [27,28]. Consequently, it is important to analyze spatial distribution of TILs subsets and macrophages separately through digital imaging and objective assessment due to their different physio-pathological effects in the tumor microenvironment [17,29,30].

It has been reported in several models that cytotoxic memory CD8^+^ T cells (CD3^+^, CD8^+^, CD45RO^+^, Granzyme B^+^) are strongly associated with a favorable clinical outcome and it has been suggested to use a combination of any two of these four markers as prognostic factors [28,31,32]. Because of technical troubles, including background noise (CD45RO) and granular staining (Granzyme B), the two easiest membrane stains, CD3 and CD8, seem to be the most reliable biomarkers [28,32].

In our series, by using a digital quantification of T cytotoxic lymphocytes, we were able to predict the DFS and OS in stage II–III intermediate/thick PCM. Interestingly, the combination of two markers (CD3^+^ and CD8^+^ T cells) in the intratumoral area significantly predicted the outcome. The fact that only intratumoral high density CD3^+^ and CD8^+^ T-cells were more significant than total density may indicate that spatial distribution and quantification plays a role in melanoma immune surveillance.

In our study, the combination of high intratumoral CD3^+^ T-cells and CD8^+^ T-cells has been shown to be a favorable predictive biomarker at multivariable analysis. Melanoma patients that were characterized by an absence or low density of intratumoral infiltration showed higher recurrence rate and shorter OS. Specifically, the combined analysis of CD8^+^ and CD3+ cells in the intratumoral location resulted in being a useful classification for the prediction of tumor recurrence in patients with intermediate/thick early stage PCM. At 5-years of follow-up, the DFS and OS for patients with high density of intratumoral CD8^+^ T-cell and CD3^+^ T-cell were 43.4% and 63.6%, respectively. Conversely, the DFS and OS rates of patients with low densities of these cells were 30.0% and 37.3% (log-rank test, DFS: *p* = 0.026).

Our results are partially in agreement with previous reports [13,33]. Piras et al. showed a significant difference in five-year survival among melanoma patient groups with high, moderate, and low CD8^+^ T-cell density [34], irrespective of density and their spatial distribution.

Importantly, we found that inflamed melanomas, with high CD3^+^ T-cells and CD8^+^ T-cells density or high CD8^+^ T-cells and CD68^+^ macrophagic infiltration, have a better prognosis when compared to those with desert melanomas characterized by low CD3^+^ and CD8^+^ T-cell density or low CD8^+^ T-cell and CD68^+^ macrophagic infiltration. This is in agreement with previous findings showing a better outcome for inflamed melanomas when compared to cold melanomas with a desert microenvironment [2].

From a clinical standpoint, our study adds information for intermediate-thick melanomas. Most of the findings on the prognostic role of TILs derive from studies that did not evaluate homogeneous cohorts of melanoma with BT >2 mm (7, 8, 14). Here, we show that the density and spatial distribution of T-cells play a role and predict the outcome of melanomas ≥2 mm.

This study presents some points of strengths: (i) patients have been enrolled and treated homogeneously in Italian centers; (ii) automatic assessment upon digital image acquisition, which allows for unbiased and rapid quantification of the immune infiltrate in immunostained tissue sections and minimizes significant user errors due to categorical rankings; and, (iii) the validation of results in an independent cohort. However, we are aware of the study limitations, including: (i) the retrospective nature of the analysis of a prospective collected cohort of patients; (ii) the relatively small series in the training and the validation cohort; and, (iii) digital analysis cannot be considered to be a standard approach for the practicing pathologists and future prospective studies are needed to better understand the adding value of this technology as compared to the standard evaluation of TME in early stages melanoma patients.

Our findings suggest that a specific preexisting profile of T cells and macrophages distribution in melanomas may predict the risk of recurrence and death with potential implications for stratification of early stages melanoma patients. This may be particularly important in stage II AJCC melanoma patients, for whom new adjuvant treatments are not available and need to be better prognosticated. Because patients with absent/low intratumoral infiltration of CD8^+^ T-cell have a statistically significant shorter DFS and OS, they may deserve further treatments to reduce the recurrence rate and ultimately progression of melanoma.

## Figures and Tables

**Table 1:** Demographic and clinical characteristics.

Variable	Training Cohort N = 100	Validation Cohort N = 74	Chi-Squared or Wilcoxon *p*-Value
**Centre**			-
University Hospital of Siena	15 (15.0)	0 (0.0)	
University of Florence	42 (42.0)	10 (13.5)	
University of Sassari/NRC	43 (43.0)	0 (0.0)	
Papa Giovanni XXIII Hospital, Bergamo	0 (0.0)	64 (86.5)	
**Age**			0.619
Mean (SD)	63.2 (16.1)	61.4 (18.6)	
Median (Q1–Q3)	66.0 (50.3–75.3)	65.8 (46.3–78.2)	
Min-Max	24.0–89.4	21.9–88.1	
**Sex**			0.486
Female	38 (38.0)	32 (43.2)	
Male	62 (62.0)	42 (56.8)	
**Tumor site**			0.962 ^a^
Limb	45 (45.0)	35 (47.9)	
Trunk	45 (45.0)	32 (43.8)	
Head/neck	8 (8.0)	6 (8.2)	
Other	2 (2.0)	0 (0.0)	
NAS	0	1	
**Histology**			0.565
Superficial spreading melanoma	49 (49.0)	40 (54.1)	
Nodular melanoma	36 (36.0)	21 (28.4)	
Other	15 (15.0)	13 (17.6)	
**Breslow thickness (mm)**			0.587
Mean (SD)	6.0 (5.3)	5.6 (4.6)	
Median (Q1–Q3)	4.3 (3.0–6.5)	4.0 (3.0–6.0)	
Min-Max	2.1–35.0	2.0–25.0	
**Mitotic rate**			0.0024
Mean (SD)	9.4 (9.1)	5.9 (4.5)	
Median (Q1–Q3)	8.0 (3.0–11.0)	5.0 (3.0–7.0)	
Min-Max	0.0–57.0	0.0–23.0	
**Clark level**			0.257
III	8 (8.1)	2 (2.7)	
IV	77 (77.8)	58 (78.4)	
V	14 (14.1)	14 (18.9)	
Missing	1	0	
**Ulceration**			0.628
No	29 (29.0)	19 (25.7)	
Yes	71 (71.0)	55 (74.3)	
**TILs**			<0.001
Absent	10 (10.0)	33 (45.2)	
Non brisk	82 (82.0)	26 (35.6)	
Brisk	8 (8.0)	14 (19.2)	
Missing	0	1	
**Stage at diagnosis**			<0.001 ^b^
I	0 (0.0)	1 (1.4)	
IB	0 (0.0)	1 (1.4)	
II	58 (58.0)	62 (83.8)	
IIA	12 (20.7)	10 (13.5)	
IIB	23 (39.7)	32 (43.2)	
IIC	23 (39.7)	20 (27.0)	
III	42 (42.0)	10 (13.5)	
IIIA	0 (0.0)	1 (1.4)	
IIIB	7 (16.7)	1 (1.4)	
IIIC	30 (71.4)	8 (10.8)	
IIID	5 (11.9)	0 (0.0)	
IV	0 (0.0)	1 (1.4)	

^a^ The *Other* and *NAS* categories were not considered for the statistical test. ^b^ The comparison was performed considering only stage II and stage III patients.

**Table 2:** Image analysis.

Density	Training Cohort N = 100	Validation Cohort N = 74	Chi-Squared or Wilcoxon *p*-Value
**CD3+**			
**Intratumoral density (cells/mm^2^)**			0.360
Mean (SD)	1543.9 (1448.4)	1202.7 (1051.8)	
Median (Q1–Q3)	997.7 (483.8–2163.5)	800.9 (451.2–1590.9)	
Min-Max	21.0–6275.8	47.8–5694.4	
Missing	19	0	
**CD3+ intratumoral density according to the median of the training cohort**			0.453
Low	40 (49.4)	41 (55.4)	
High	41 (50.6)	33 (44.6)	
Missing	19	0	
**CD3+ peritumoral IN density (cells/mm^2^)**			0.014
Mean (SD)	2281.1 (1483.6)	1765.0 (1492.4)	
Median (Q1–Q3)	2146.9 (1084.5–3162.3)	1334.0 (775.0–2334.8)	
Min-Max	61.4–6619.7	0.0–8655.1	
Missing	11	3	
**CD3+ peritumoral IN density according to the median of the training cohort**			0.021
Low	44 (49.4)	48 (67.6)	
High	45 (50.6)	23 (32.4)	
Missing	11	3	
**CD3+ peritumoral OUT density (cells/mm^2^)**			<0.001
Mean (SD)	1916.9 (1363.2)	379.5 (335.5)	
Median (Q1–Q3)	1611.6 (796.2–2810.5)	263.3 (146.3–483.5)	
Min-Max	84.6–5300.5	27.6–1788.6	
Missing	11	3	
**CD3+ peritumoral OUT density according to the median of the training cohort**			<0.001
Low	44 (49.4)	70 (98.6)	
High	45 (50.6)	1 (1.4)	
Missing	11	3	
**CD4+ intratumoral density (cells/mm^2^)**			0.340
Mean (SD)	1675.2 (1379.9)	1532.3 (1422.1)	
Median (Q1–Q3)	1421.9 (649.5–2333.5)	1224.8 (551.6–2166.8)	
Min-Max	0.0–7304.6	11.4–8665.0	
Missing	4		
**CD4+ intratumoral density according to the median of the training cohort**			0.293
Low	48 (50.0)	43 (58.1)	
High	48 (50.0)	31 (41.9)	
Missing	4	0	
**CD4+ peritumoral IN density (cells/mm^2^)**			0.005
Mean (SD)	2622.3 (1662.8)	1882.2 (1247.8)	
Median (Q1–Q3)	2384.0 (1264.5–3727.1)	1714.5 (854.8–2580.0)	
Min-Max	0.4–6760.9	16.7–5601.2	
Missing	11	4	
**CD4+ peritumoral IN density according to the median of the training cohort**			0.005
Low	44 (49.4)	50 (71.4)	
High	45 (50.6)	20 (28.6)	
Missing	11	4	
**CD4+ peritumoral OUT density (cells/mm^2^)**			<0.001
Mean (SD)	2177.9 (1467.5)	601.4 (440.3)	
Median (Q1–Q3)	1965.3 (1058.0–3124.4)	495.1 (254.3–804.8)	
Min-Max	0.0–6137.5	24.4–1979.4	
Missing	11	4	
**CD4+ peritumoral OUT density according to the median of the training cohort**			<0.001
Low	44 (49.4)	69 (98.6)	
High	45 (50.6)	1 (1.4)	
Missing	11	4	
**CD8+ intratumoral density (cells/mm^2^)**			0.823
Mean (SD)	868.8 (1028.3)	751.4 (719.6)	
Median (Q1–Q3)	553.8 (160.7–1181.2)	441.9 (258.1–1187.4)	
Min-Max	13.1–6559.4	32.0–3809.9	
MIssing	5	0	
**CD8+ intratumoral density according to the median of the training cohort**			0.142
Low	47 (49.5)	45 (60.8)	
High	48 (50.5)	29 (39.2)	
Missing	5	0	
**CD8+ peritumoral IN density (cells/mm^2^)**			0.090
Mean (SD)	1429.1 (1335.5)	1054.4 (1062.0)	
Median (Q1–Q3)	1032.2 (447.1–2266.7)	654.9 (320.8–1398.9)	
Min-Max	33.2–5911.3	17.6–5334.9	
Missing	11	3	
**CD8+ peritumoral IN density according to the median of the training cohort**			0.034
Low	44 (49.4)	47 (66.2)	
High	45 (50.6)	24 (33.8)	
Missing	11	3	
**CD8+ peritumoral OUT density (cells/mm^2^)**			<0.001
Mean (SD)	1119.8 (1122.1)	227.0 (287.3)	
Median (Q1–Q3)	646.2 (358.1–1406.7)	154.5 (80.0–231.5)	
Min-Max	4.8–5088.9	5.3–1651.2	
Missing	11	3	
**CD8+ peritumoral OUT density according to the median of the training cohort**			<0.001
Low	44 (49.4)	67 (94.4)	
High	45 (50.6)	4 (5.6)	
Missing	11	3	
**CD68+ intratumoral density (cells/mm^2^)**			0.015
Mean (SD)	367.2 (398.5)	583.7 (633.1)	
Median (Q1–Q3)	248.1 (95.6–488.8)	363.8 (172.6–763.1)	
Min-Max	4.8–1981.5	1.7–2958.6	
Missing	6	0	
**CD68+ intratumoral density according to the median of the training cohort**			0.296
Low	47 (50.0)	31 (41.9)	
High	47 (50.0)	43 (58.1)	
Missing	6	0	
**CD68+ peritumoral IN density (cells/mm^2^)**			0.281
Mean (SD)	469.7 (494.6)	611.7 (658.4)	
Median (Q1–Q3)	264.3 (128.9–661.9)	459.7 (144.1–773.6)	
Min-Max	0.0–2589.4	1.3–3022.2	
Missing	11	3	
**CD68+ peritumoral IN density according to the median of the training cohort**			0.104
Low	44 (49.4)	26 (36.6)	
High	45 (50.6)	45 (63.4)	
Missing	11	3	
**CD68+ peritumoral OUT density (cells/mm^2^)**			<0.001
Mean (SD)	337.9 (312.6)	88.7 (120.3)	
Median (Q1–Q3)	243.4 (116.7–476.0)	51.9 (5.9–136.7)	
Min-Max	2.9–1775.6	0.0–588.2	
Missing	11	3	
**CD68+ peritumoral OUT density according to the median of the training cohort**			<0.001
Low	45 (50.6)	67 (94.4)	
High	44 (49.4)	4 (5.6)	
Missing	11	3	
**CD163+ intratumoral density (cells/mm^2^)**			-
Mean (SD)	1188.9 (1073.4)	-	
Median (Q1–Q3)	757.6 (481.2–1580.8)	-	
Min-Max	18.8–5017.9	-	
Missing	4	-	
**CD163+ peritumoral IN density (cells/mm^2^)**			-
Mean (SD)	1472.6 (1090.4)	-	
Median (Q1–Q3)	1205.6 (608.6–2033.9)	-	
Min-Max	41.4–5147.3	-	
Missing	11	-	
**CD163+ peritumoral OUT density (cells/mm^2^)**			-
Mean (SD)	1061.4 (691.7)	-	
Median (Q1–Q3)	878.3 (548.9–1388.2)	-	
Min-Max	101.4–3456.6	-	
Missing	11	-	
**FOXP3 intratumoral density (cells/mm^2^)**			-
Mean (SD)	528.5 (1297.7)	-	
Median (Q1–Q3)	40.5 (2.2–315.3)	-	
Min-Max	0.0–6794.9	-	
Missing	4	-	
**FOXP3 peritumoral IN density (cells/mm^2^)**			-
Mean (SD)	430.1 (1031.0)	-	
Median (Q1–Q3)	69.2 (1.5–409.2)	-	
Min-Max	0.0–7104.4	-	
Missing	11	-	
**FOXP3 peritumoral OUT density (cells/mm^2^)**			-
Mean (SD)	292.4 (602.4)	-	
Median (Q1–Q3)	40.3 (0.5–306.1)	-	
Min-Max	0.0–2898.8	-	
Missing	11	-	
**PD1 intratumoral density (cells/mm^2^)**			-
Mean (SD)	440.4 (604.6)	-	
Median (Q1–Q3)	253.5 (57.9–507.5)	-	
Min-Max	3.7–3038.4	-	
Missing	4	-	
**PD1 peritumoral IN density (cells/mm^2^)**			-
Mean (SD)	806.4 (959.3)	-	
Median (Q1–Q3)	512.3 (164.9–1124.6)	-	
Min-Max	7.2–5068.7	-	
Missing	11	-	
**PD1 peritumoral OUT density (cells/mm^2^)**			-
Mean (SD)	549.9 (678.0)	-	
Median (Q1–Q3)	361.4 (160.9–654.2)	-	
Min-Max	6.2–4411.1	-	
Missing	11	-	
**PD-L1 intratumoral density (cells/mm^2^)**			-
Mean (SD)	358.5 (872.7)	-	
Median (Q1–Q3)	39.9 (11.9–260.2)	-	
Min-Max	0.1–6251.2	-	
Missing	4	-	
**PD-L1 peritumoral IN density (cells/mm^2^)**			-
Mean (SD)	331.3 (685.1)	-	
Median (Q1–Q3)	52.0 (8.8–257.5)	-	
Min-Max	0.0–3501.0	-	
Missing	11	-	
**PD-L1 peritumoral OUT density (cells/mm^2^)**			-
Mean (SD)	108.0 (192.5)	-	
Median (Q1–Q3)	30.1 (9.4–124.0)	-	
Min-Max	0.0–919.1	-	
Missing	11	-	

**Table 3:** Effect of biomarkers density on disease free survival and overall survival in the training cohort. Univariable and multivariable Cox proportional hazard models.

	DISEASE FREE SURVIVAL				OVERALL SURVIVAL			
	Univariable Analysis		Multivariable Analysis		Univariable Analysis		Multivariable Analysis	
	HR (95% CI)	*p*-Value	HR (95% CI)	*p*-Value	HR (95% CI)	*p*-Value	HR (95% CI)	*p*-Value
**CD3+**								
**Intratumoral** *(100 cell/mm2 increase)*	0.98 (0.96–1.00)	0.060	0.98 (0.96–1.00)	0.059	0.98 (0.96–1.00)	0.121	0.98 (0.96–1.00)	0.074
**Intratumoral***(high* vs. *low)*	0.59 (0.35–1.00)	0.050 *	0.59 (0.33–1.05)	0.072	0.72 (0.41–1.27)	0.256	0.66 (0.36–1.20)	0.173
**Peritumoral IN** *(100 cell/mm2 increase)*	0.98 (0.96–1.00)	0.072	0.98 (0.97–1.00)	0.090	0.98 (0.96–1.00)	0.112	0.99 (0.96–1.01)	0.179
**Peritumoral IN***(high* vs. *low)*	0.75 (0.44–1.28)	0.290	0.78 (0.46–1.33)	0.361	0.75 (0.42–1.34)	0.338	0.76 (0.43–1.37)	0.365
**Peritumoral OUT** *(100 cell/mm2 increase)*	0.98 (0.96–1.00)	0.120	0.98 (0.96–1.00)	0.055	0.98 (0.96–1.01)	0.183	0.98 (0.95–1.00)	0.044 *
**Peritumoral OUT***(high* vs. *low)*	0.69 (0.40–1.18)	0.172	0.62 (0.36–1.07)	0.086	0.80 (0.45–1.44)	0.459	0.64 (0.35–1.15)	0.137
**CD4+**								
**Intratumoral** *(100 cell/mm2 increase)*	0.98 (0.95–1.00)	0.032 *	0.98 (0.95–1.00)	0.041 *	0.98 (0.95–1.00)	0.073	0.98 (0.95–1.00)	0.104
**Intratumoral***(high* vs. *low)*	0.67 (0.41–1.09)	0.109	0.70 (0.43–1.16)	0.166	0.67 (0.39–1.17)	0.159	0.75 (0.43–1.31)	0.318
**Peritumoral IN** *(100 cell/mm2 increase)*	0.98 (0.96–1.00)	0.056	0.99 (0.97–1.01)	0.196	0.98 (0.96–1.00)	0.086	0.99 (0.97–1.01)	0.296
**Peritumoral IN***(high* vs. *low)*	0.71 (0.42–1.20)	0.201	0.83 (0.48–1.44)	0.510	0.79 (0.45–1.41)	0.434	0.97 (0.54–1.75)	0.925
**Peritumoral OUT** *(100 cell/mm2 increase)*	0.98 (0.97–1.00)	0.123	0.98 (0.96–1.00)	0.096	0.99 (0.97–1.01)	0.412	0.98 (0.96–1.01)	0.175
**Peritumoral OUT***(high* vs. *low)*	0.74 (0.44–1.26)	0.270	0.70 (0.40–1.22)	0.210	0.80 (0.45–1.42)	0.443	0.64 (0.35–1.18)	0.153
**CD8+**								
**Intratumoral** *(100 cell/mm2 increase)*	0.98 (0.95–1.01)	0.108	0.99 (0.96–1.02)	0.387	0.96 (0.92–1.00)	0.050 *	0.98 (0.94–1.01)	0.219
**Intratumoral***(high* vs. *low)*	0.70 (0.42–1.15)	0.160	0.74 (0.45–1.22)	0.241	0.57 (0.32–0.99)	0.048 *	0.64 (0.36–1.13)	0.122
**Peritumoral IN** *(100 cell/mm2 increase)*	0.99 (0.97–1.01)	0.287	1.00 (0.97–1.02)	0.693	0.98 (0.95–1.01)	0.112	0.99 (0.96–1.02)	0.451
**Peritumoral IN***(high* vs. *low)*	0.90 (0.53–1.52)	0.684	1.05 (0.60–1.81)	0.872	0.73 (0.41–1.31)	0.293	0.87 (0.47–1.60)	0.657
**Peritumoral OUT** *(100 cell/mm2 increase)*	0.98 (0.95–1.01)	0.157	0.98 (0.95–1.01)	0.171	0.98 (0.94–1.01)	0.152	0.97 (0.94–1.01)	0.151
**Peritumoral OUT***(high* vs. *low)*	0.95 (0.56–1.62)	0.853	0.98 (0.57–1.69)	0.947	0.75 (0.42–1.35)	0.336	0.74 (0.41–1.35)	0.323
**CD68+**								
**Intratumoral** *(100 cell/mm2 increase)*	1.00 (0.99–1.01)	0.775	1.00 (0.99–1.01)	0.934	1.00 (0.99–1.00)	0.322	1.00 (0.99–1.00)	0.328
**Intratumoral***(high* vs. *low)*	0.86 (0.52–1.42)	0.547	0.78 (0.46–1.31)	0.349	0.51 (0.29–0.92)	0.025 *	0.52 (0.29–0.95)	0.033 *
**Peritumoral IN** *(100 cell/mm2 increase)*	1.00 (0.99–1.00)	0.366	1.00 (0.99–1.00)	0.553	1.00 (0.99–1.00)	0.429	1.00 (0.99–1.01)	0.588
**Peritumoral IN***(high* vs. *low)*	0.83 (0.48–1.40)	0.478	0.79 (0.46–1.37)	0.408	0.75 (0.42–1.36)	0.345	0.73 (0.40–1.32)	0.298
**Peritumoral OUT** *(100 cell/mm2 increase)*	1.00 (1.00–1.01)	0.451	1.00 (1.00–1.01)	0.368	1.00 (0.99–1.01)	0.862	1.00 (0.99–1.01)	0.864
**Peritumoral OUT***(high* vs. *low)*	1.10 (0.65–1.86)	0.725	1.02 (0.58–1.79)	0.945	0.85 (0.47–1.51)	0.574	0.70 (0.38–1.30)	0.259
**CD163+**								
**Intratumoral** *(100 cell/mm2 increase)*	0.97 (0.95–1.00)	0.046 *	0.98 (0.96–1.00)	0.070	0.97 (0.95–1.00)	0.062	0.98 (0.95–1.00)	0.094
**Intratumoral***(high* vs. *low)*	0.68 (0.41–1.13)	0.135	0.85 (0.51–1.44)	0.548	0.59 (0.33–1.03)	0.063	0.77 (0.43–1.39)	0.387
**Peritumoral IN** *(100 cell/mm2 increase)*	0.98 (0.95–1.00)	0.093	0.98 (0.96–1.01)	0.127	0.98 (0.95–1.01)	0.170	0.99 (0.96–1.01)	0.275
**Peritumoral IN***(high* vs. *low)*	0.53 (0.31–0.90)	0.019 *	0.56 (0.32–0.99)	0.047 *	0.57 (0.32–1.03)	0.064	0.64 (0.35–1.19)	0.158
**Peritumoral OUT** *(100 cell/mm2 increase)*	0.97 (0.93–1.01)	0.198	0.97 (0.93–1.01)	0.123	0.98 (0.94–1.03)	0.393	0.97 (0.94–1.01)	0.206
**Peritumoral OUT***(high* vs. *low)*	0.69 (0.41–1.18)	0.174	0.73 (0.43–1.25)	0.248	0.70 (0.39–1.26)	0.236	0.70 (0.39–1.26)	0.231
**FOXP3**								
**Intratumoral** *(10 cell/mm2 increase)*	1.00 (1.00–1.00)	0.359	1.00 (1.00–1.00)	0.804	1.00 (1.00–1.00)	0.050	1.00 (1.00–1.00)	0.329
**Intratumoral***(high* vs. *low)*	0.84 (0.51–1.38)	0.498	0.66 (0.39–1.10)	0.112	1.25 (0.72–2.16)	0.432	0.94 (0.53–1.66)	0.827
**Peritumoral IN** *(10 cell/mm2 increase)*	1.00 (1.00–1.00)	0.713	1.00 (1.00–1.00)	0.377	1.00 (1.00–1.00)	0.565	1.00 (1.00–1.00)	0.956
**Peritumoral IN***(high* vs. *low)*	0.72 (0.42–1.21)	0.216	0.59 (0.34–1.02)	0.057	0.81 (0.46–1.44)	0.477	0.63 (0.35–1.14)	0.126
**Peritumoral OUT** *(10 cell/mm2 increase)*	1.00 (0.99–1.00)	0.365	1.00 (0.99–1.00)	0.126	1.00 (1.00–1.00)	0.950	1.00 (0.99–1.00)	0.295
**Peritumoral OUT***(high* vs. *low)*	0.80 (0.47–1.35)	0.401	0.66 (0.39–1.13)	0.130	0.87 (0.49–1.55)	0.642	0.61 (0.34–1.12)	0.109
**PD1**								
**Intratumoral** *(100 cell/mm2 increase)*	0.98 (0.93–1.02)	0.287	1.00 (0.95–1.05)	0.916	0.97 (0.91–1.02)	0.227	1.00 (0.94–1.06)	0.893
**Intratumoral***(high* vs. *low)*	0.84 (0.51–1.38)	0.492	0.96 (0.57–1.62)	0.878	0.58 (0.33–1.03)	0.061	0.63 (0.35–1.12)	0.117
**Peritumoral IN** *(100 cell/mm2 increase)*	0.97 (0.94–1.01)	0.116	0.98 (0.95–1.02)	0.308	0.97 (0.93–1.01)	0.116	0.98 (0.94–1.02)	0.376
**Peritumoral IN***(high* vs. *low)*	0.62 (0.36–1.06)	0.078	0.70 (0.40–1.22)	0.210	0.53 (0.29–0.97)	0.039 *	0.59 (0.32–1.10)	0.095
**Peritumoral OUT** *(100 cell/mm2 increase)*	0.97 (0.93–1.02)	0.262	0.98 (0.94–1.03)	0.447	0.97 (0.92–1.02)	0.259	0.97 (0.92–1.03)	0.305
**Peritumoral OUT***(high* vs. *low)*	0.92 (0.54–1.55)	0.747	1.04 (0.59–1.82)	0.894	0.80 (0.45–1.43)	0.456	0.86 (0.47–1.59)	0.638
**PD-L1**								
**Intratumoral** *(10 cell/mm2 increase)*	1.00 (1.00–1.00)	0.764	1.00 (1.00–1.00)	0.823	1.00 (1.00–1.00)	0.847	1.00 (1.00–1.00)	0.624
**Intratumoral***(high* vs. *low)*	0.90 (0.54–1.49)	0.678	1.03 (0.62–1.74)	0.897	0.83 (0.47–1.46)	0.514	0.98 (0.55–1.75)	0.957
**Peritumoral IN** *(10 cell/mm2 increase)*	0.99 (0.99–1.00)	0.069	1.00 (0.99–1.00)	0.128	0.99 (0.98–1.00)	0.085	0.99 (0.99–1.00)	0.154
**Peritumoral IN***(high* vs. *low)*	0.70 (0.41–1.19)	0.188	0.76 (0.44–1.31)	0.327	0.51 (0.28–0.93)	0.027 *	0.58 (0.31–1.07)	0.079
**Peritumoral OUT** *(10 cell/mm2 increase)*	0.99 (0.97–1.00)	0.123	0.98 (0.97–1.00)	0.098	0.99 (0.97–1.01)	0.241	0.98 (0.96–1.00)	0.128
**Peritumoral OUT***(high* vs. *low)*	0.94 (0.55–1.61)	0.830	0.92 (0.53–1.60)	0.772	0.94 (0.52–1.69)	0.839	0.80 (0.44–1.46)	0.461

**Note.** Multivariable models adjusted for Breslow thickness, ulceration and stage. * Significant *p*-value at 0.05 level.

## Data Availability

The data presented in this study are available on request from the corresponding author.

## Supplementary Materials

The following are available online at https://www.mdpi.com/2073-4409/10/2/422/s1, Figure S1: Representative immunohistochemical images of CD3 (A), CD4 (B), CD8 (C) and CD68 (D) in melanoma tissue, Figure S2: Representative images of melanoma tissue with low CD8 (A) and low CD68 (B), Table S1: Biomarkers associations in the training cohort. Spearman correlation index., Table S2: Associations between biomarkers and clinical characteristics in the training cohort. Chi-square and Wilcoxon test, Table S3: Spatial distribution of biomarkers, Table S4: Biomarkers associations in the validation cohort. Spearman correlation index, Table S5: Associations between biomarkers and clinical characteristics in the validation cohort. Chi-square and Wilcoxon test, Table S6: Effect of biomarkers density on disease free survival and overall survival in the validation cohort. Univariable and multivariable Cox proportional hazard models.
