# Supplementary material for: Digital Immunophenotyping Predicts Disease Free and Overall Survival in Early Stage Melanoma Patients
Source: Cells. 2021 Feb 17;10(2):422. doi: 10.3390/cells10020422 (PMC7922113; doi:10.3390/cells10020422)
Supplement: Supplementary file 1 [file cells-10-00422-s001.pdf]

Supplementary Figures

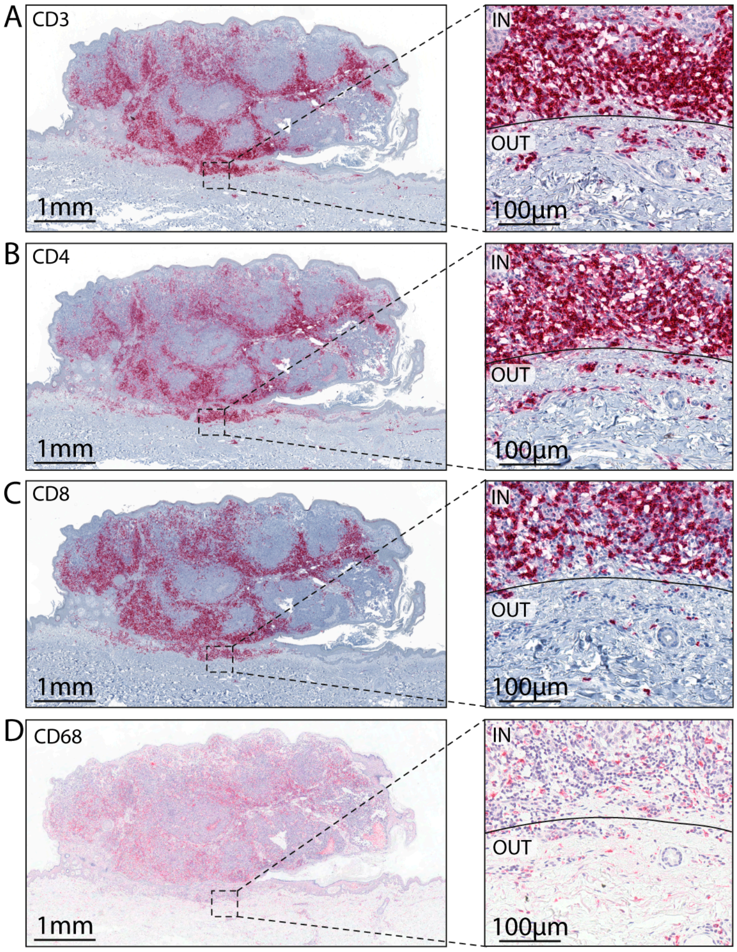

Figure S1. Representative immunohistochemical images of CD3 (A), CD4 (B), CD8 (C) and CD68 (D) in melanoma tissue. IN, indicates the inner portion of the peritumoral area, OUT, indicates the outer portion of the peritumoral area.

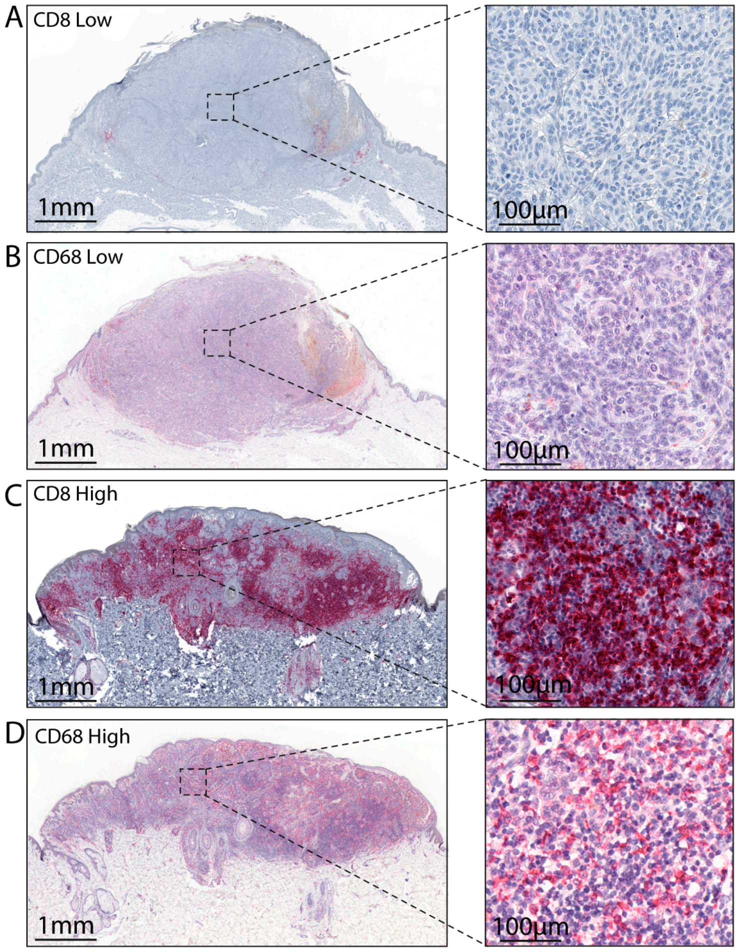

Figure S2. Representative images of melanoma tissue with low CD8 (A) and low CD68 (B). Representative images of melanoma tissue with high CD8 (C) and high CD68 (D).

Supplementary Tables

Table S1. Biomarkers associations in the training cohort. Spearman correlation index.

	**CD3+**			**CD4+**			**CD8+**			**CD68+**			**CD163+**			**FOXP3**			**PD1**			**PD-L1**		
	**Intra**	**IN**	**OUT**	**Intra**	**IN**	**OUT**	**Intra**	**IN**	**OUT**	**Intra**	**IN**	**OUT**	**Intra**	**IN**	**OUT**	**Intra**	**IN**	**OUT**	**Intra**	**IN**	**OUT**	**Intra**	**IN**	**OUT**
**CD3+**																								
**Intra**	1	**0.80**	0.60	**0.75**	0.56	0.48	**0.71**	0.60	0.52	0.31	0.31	0.14	0.44	0.32	0.30	0.50	0.41	0.34	0.57	0.51	0.35	0.42	0.40	0.26
**IN**		1	**0.75**	0.69	**0.77**	0.59	**0.71**	**0.80**	**0.73**	0.38	0.45	0.28	0.45	0.53	0.46	0.41	0.45	0.43	0.59	0.67	0.49	0.46	0.60	0.44
**OUT**			1	0.45	0.62	0.69	0.43	0.57	**0.80**	0.34	0.45	0.38	0.32	0.46	0.45	0.33	0.41	0.49	0.49	0.60	0.52	0.38	0.52	0.47
**CD4+**																								
**Intra**				1	**0.76**	0.55	0.67	0.59	0.50	0.36	0.40	0.18	0.59	0.46	0.42	0.38	0.34	0.28	0.57	0.52	0.40	0.62	0.61	0.42
**IN**					1	**0.78**	0.54	**0.72**	0.69	0.28	0.45	0.32	0.46	0.56	0.51	0.37	0.42	0.43	0.50	0.63	0.46	0.51	0.67	0.53
**OUT**						1	0.31	0.47	0.68	0.25	0.45	0.46	0.28	0.45	0.55	0.41	0.46	0.52	0.36	0.47	0.44	0.40	0.56	0.58
**CD8+**																								
**Intra**							1	**0.85**	0.65	0.50	0.40	0.40	0.56	0.44	0.40	0.13	0.16	0.15	**0.80**	**0.73**	0.59	0.61	0.56	0.40
**IN**								1	**0.84**	0.45	0.51	0.53	0.44	0.52	0.45	0.22	0.29	0.31	**0.71**	**0.82**	0.63	0.55	0.68	0.51
**OUT**									1	0.12	0.25	0.40	0.34	0.53	0.53	0.25	0.34	0.42	0.63	**0.76**	**0.70**	0.53	0.67	0.57
**CD68+**																								
**Intra**										1	**0.80**	0.61	0.29	0.28	0.38	-0.09	0.00	0.01	0.55	0.47	0.49	0.43	0.46	0.34
**IN**											1	**0.78**	0.24	0.38	0.50	0.03	0.14	0.19	0.51	0.54	0.56	0.39	0.52	0.39
**OUT**												1	0.02	0.22	0.44	0.14	0.20	0.26	0.25	0.30	0.45	0.17	0.33	0.35
**CD163+**																								
**Intra**													1	**0.82**	0.61	0.16	0.18	0.18	0.58	0.42	0.32	0.61	0.52	0.34
**IN**														1	**0.80**	0.21	0.35	0.38	0.47	0.45	0.38	0.47	0.60	0.45

	CD3+			CD4+			CD8+			CD68+			CD163+			FOXP3			PD1			PD-L1		
	Intra	IN	OUT	Intra	IN	OUT	Intra	IN	OUT	Intra	IN	OUT	Intra	IN	OUT	Intra	IN	OUT	Intra	IN	OUT	Intra	IN	OUT
**OUT**															1	0.31	0.44	0.46	0.42	0.40	0.44	0.44	0.61	0.58
**FOXP3**																								
**Intra**																1	**0.91**	**0.84**	0.02	0.11	0.04	0.28	0.40	0.43
**IN**																	1	**0.95**	0.07	0.23	0.11	0.25	0.46	0.47
**OUT**																		1	0.10	0.26	0.18	0.24	0.47	0.49
**PD1**																								
**Intra**																			1	**0.86**	**0.74**	0.65	0.60	0.43
**IN**																				1	**0.77**	0.56	0.65	0.48
**OUT**																					1	0.47	0.54	0.51
**PD-L1**																								
**Intra**																						1	**0.83**	0.69
**IN**																							1	**0.85**
**OUT**																								1

Legend. Intra: intratumoral. IN: peritumoral IN. OUT: peritumoral OUT.

Table S2. Associations between biomarkers and clinical characteristics in the training cohort. Chi-square and Wilcoxon test.

	**Stage at diagnosis** – n (%)			**Breslow thickness (mm)**		**Ulceration** – n (%)		
	**Stage II**	**Stage III**	**P-value**	**Median (Q1-Q3)**	**P-value**	**Absent**	**Present**	**P-value**
**Intratumoral**			0.432		0.533			0.666
**Low CD3+**	23 (53.5)	17 (44.7)		5.0 (3.0-6.8)		10 (45.5)	30 (50.8)	
**High CD3+**	20 (46.5)	21 (55.3)		3.8 (3.0-6.5)		12 (54.5)	29 (49.2)	
**Peritumoral IN**			0.603		0.749			0.865
**Low CD3+**	24 (47.1)	20 (52.6)		4.0 (2.7-6.0)		12 (48.0)	32 (50.0)	
**High CD3+**	27 (52.9)	18 (47.4)		4.0 (3.1-6.0)		13 (52.0)	32 (50.0)	
**Peritumoral OUT**			0.927		0.318			0.086
**Low CD3+**	25 (49.0)	19 (50.0)		3.5 (2.7-6.0)		16 (64.0)	28 (43.8)	
**High CD3+**	26 (51.0)	19 (50.0)		4.0 (3.2-6.0)		9 (36.0)	36 (56.3)	
**Intratumoral**			0.536		0.684			0.496
**Low CD4+**	26 (47.3)	22 (53.7)		4.7 (3.0-6.4)		12 (44.4)	36 (52.2)	
**High CD4+**	29 (52.7)	19 (46.3)		3.8 (2.9-6.5)		15 (55.6)	33 (47.8)	
**Peritumoral IN**			0.071		0.613			0.865
**Low CD4+**	21 (41.2)	23 (60.5)		4.0 (2.7-6.0)		12 (48.0)	32 (50.0)	
**High CD4+**	30 (58.8)	15 (39.5)		4.0 (3.0-6.0)		13 (52.0)	32 (50.0)	
**Peritumoral OUT**			0.603		0.215			**0.029***
**Low CD4+**	24 (47.1)	20 (52.6)		3.5 (2.7-6.0)		17 (68.0)	27 (42.2)	
**High CD4+**	27 (52.9)	18 (47.4)		4.3 (3.2-6.3)		8 (32.0)	37 (57.8)	
**Intratumoral**			0.124		0.381			0.871
**Low CD8+**	23 (42.6)	24 (58.5)		5.0 (2.7-8.0)		13 (48.1)	34 (50.0)	
**High CD8+**	31 (57.4)	17 (41.5)		3.8 (3.0-6.0)		14 (51.9)	34 (50.0)	
**Peritumoral IN**			**0.025***		0.690			0.865
**Low CD8+**	20 (39.2)	24 (63.2)		4.0 (2.7-6.2)		12 (48.0)	32 (50.0)	
**High CD8+**	31 (60.8)	14 (36.8)		4.0 (3.1-6.0)		13 (52.0)	32 (50.0)	
**Peritumoral OUT**			0.168		0.438			0.439

	Stage at diagnosis – n (%)			Breslow thickness (mm)		Ulceration – n (%)		
	Stage II	Stage III	P-value	Median (Q1-Q3)	P-value	Absent	Present	P-value
**Low CD8+**	22 (43.1)	22 (57.9)		3.5 (2.7-6.2)		14 (56.0)	30 (46.9)	
**High CD8+**	29 (56.9)	16 (42.1)		4.3 (3.1-6.0)		11 (44.0)	34 (53.1)	
**Intratumoral**			0.095		0.323			0.254
**Low CD68+**	23 (42.6)	24 (60.0)		3.8 (2.6-6.5)		11 (40.7)	36 (53.7)	
**High CD68+**	31 (57.4)	16 (40.0)		4.5 (3.3-6.0)		16 (59.3)	31 (46.3)	
**Peritumoral IN**			0.343		0.380			0.763
**Low CD68+**	23 (45.1)	21 (55.3)		3.6 (2.4-6.4)		13 (52.0)	31 (48.4)	
**High CD68+**	28 (54.9)	17 (44.7)		4.0 (3.2-5.0)		12 (48.0)	33 (51.6)	
**Peritumoral OUT**			0.444		0.075			0.521
**Low CD68+**	24 (47.1)	21 (55.3)		3.5 (2.6-5.9)		14 (56.0)	31 (48.4)	
**High CD68+**	27 (52.9)	17 (44.7)		4.5 (3.3-6.2)		11 (44.0)	33 (51.6)	
**Intratumoral**			**0.023***		0.196			0.496
**Low CD163+**	22 (40.0)	26 (63.4)		5.0 (3.0-7.0)		12 (44.4)	36 (52.2)	
**High CD163+**	33 (60.0)	15 (36.6)		3.9 (2.9-6.0)		15 (55.6)	33 (47.8)	
**Peritumoral IN**			0.105		0.506			0.865
**Low CD163+**	22 (43.1)	23 (60.5)		4.8 (2.7-6.0)		13 (52.0)	32 (50.0)	
**High CD163+**	29 (56.9)	15 (39.5)		3.9 (3.0-5.4)		12 (48.0)	32 (50.0)	
**Peritumoral OUT**			0.343		0.582			0.439
**Low CD163+**	23 (45.1)	21 (55.3)		3.6 (2.5-6.0)		14 (56.0)	30 (46.9)	
**High CD163+**	28 (54.9)	17 (44.7)		4.0 (3.2-6.0)		11 (44.0)	34 (53.1)	
**Intratumoral**			0.063		0.111			0.496
**Low FOXP3**	32 (58.2)	16 (39.0)		3.9 (2.5-6.0)		15 (55.6)	33 (47.8)	
**High FOXP3**	23 (41.8)	25 (61.0)		4.7 (3.3-6.8)		12 (44.4)	36 (52.2)	
**Peritumoral IN**			0.105		0.175			0.865
**Low FOXP3**	29 (56.9)	15 (39.5)		3.7 (2.5-5.7)		12 (48.0)	32 (50.0)	
**High FOXP3**	22 (43.1)	23 (60.5)		4.2 (3.2-6.4)		13 (52.0)	32 (50.0)	

	Stage at diagnosis – n (%)			Breslow thickness (mm)		Ulceration – n (%)		
	**Stage II**	**Stage III**	**P-value**	**Median (Q1-Q3)**	**P-value**	**Absent**	**Present**	**P-value**
**Peritumoral OUT**			0.232		**0.021***			0.763
**Low FOXP3**	28 (54.9)	16 (42.1)		3.3 (2.4-5.3)		13 (52.0)	31 (48.4)	
**High FOXP3**	23 (45.1)	22 (57.9)		4.5 (3.4-6.4)		12 (48.0)	33 (51.6)	
**Intratumoral**			0.063		0.313			0.820
**Low PD1**	23 (41.8)	25 (61.0)		5.0 (3.0-7.5)		13 (48.1)	35 (50.7)	
**High PD1**	32 (58.2)	16 (39.0)		4.0 (2.9-5.9)		14 (51.9)	34 (49.3)	
**Peritumoral IN**			**0.013***		0.709			0.266
**Low PD1**	20 (39.2)	25 (65.8)		4.2 (2.8-6.5)		15 (60.0)	30 (46.9)	
**High PD1**	31 (60.8)	13 (34.2)		3.9 (2.9-5.9)		10 (40.0)	34 (53.1)	
**Peritumoral OUT**			**0.008***		0.347			0.213
**Low PD1**	19 (37.3)	25 (65.8)		3.7 (2.5-6.2)		15 (60.0)	29 (45.3)	
**High PD1**	32 (62.7)	13 (34.2)		4.0 (3.2-6.0)		10 (40.0)	35 (54.7)	
**Intratumoral**			0.063		0.486			0.496
**Low PD-L1**	23 (41.8)	25 (61.0)		4.4 (2.5-6.8)		15 (55.6)	33 (47.8)	
**High PD-L1**	32 (58.2)	16 (39.0)		4.3 (3.3-6.0)		12 (44.4)	36 (52.2)	
**Peritumoral IN**			**0.008***		0.345			0.763
**Low PD-L1**	19 (37.3)	25 (65.8)		3.6 (2.4-6.3)		13 (52.0)	31 (48.4)	
**High PD-L1**	32 (62.7)	13 (34.2)		4.0 (3.3-6.0)		12 (48.0)	33 (51.6)	
**Peritumoral OUT**			0.736		0.511			0.113
**Low PD-L1**	25 (49.0)	20 (52.6)		3.8 (2.6-6.0)		16 (64.0)	29 (45.3)	
**High PD-L1**	26 (51.0)	18 (47.4)		4.0 (3.2-6.0)		9 (36.0)	35 (54.7)	

* Significant p-value at 0.05 level.

Table S3. Spatial distribution of biomarkers.

Density (cells/mm^2) - Mean (SD)	Training cohort N=100					Validation cohort N=74				
	Intratumoral	Peritumoral IN	Peritumoral OUT	Overall F-test p-value	T-test p-value	Intratumoral	Peritumoral IN	Peritumoral OUT	Overall F-test p-value	T-test p-value
**CD3+**	1543.9 (1448.4)	2281.1 (1483.6)	1916.9 (1363.2)	<0.001	A:0.001<br>B:0.087<br>C:0.090	1202.7 (1051.8)	1765.0 (1492.4)	379.5 (335.5)	<0.0001	A:0.010<br>B:<0.001<br>C:<0.001
**CD4+**	1675.2 (1379.9)	2622.3 (1662.8)	2177.9 (1467.5)	<0.001	A:<0.001<br>B:0.017<br>C:0.060	1532.3 (1422.1)	1882.2 (1247.8)	601.4 (440.3)	<0.0001	A:0.120<br>B:<0.001<br>C:<0.001
**CD8+**	868.8 (1028.3)	1429.1 (1335.5)	1119.8 (1122.1)	<0.001	A:0.002<br>B:0.115<br>C:0.096	751.4 (719.6)	1054.4 (1062.0)	227.0 (287.3)	<0.0001	A:0.047<br>B:<0.001<br>C:<0.001
**CD68+**	367.2 (398.5)	469.7 (494.6)	337.9 (312.6)	0.002	A:0.126<br>B:0.580<br>C:0.035	583.7 (633.1)	611.7 (658.4)	88.7 (120.3)	<0.0001	A:0.794<br>B:<0.001<br>C:<0.001
**CD163+**	1188.9 (1073.4)	1472.6 (1090.4)	1061.4 (691.7)	<0.001	A: 0.076<br>B:0.335<br>C: 0.003					
**FOXP3**	528.5 (1297.7)	430.1 (1031.0)	292.4 (602.4)	0.098	-					

A: Intratumoral vs Peritumoral IN. B: Intratumoral vs Peritumoral OUT. C: Peritumoral IN vs Peritumoral OUT

Table S4. Biomarkers associations in the validation cohort. Spearman correlation index.

	CD3+			CD4+			CD8+			CD68+		
	Intra	IN	OUT	Intra	IN	OUT	Intra	IN	OUT	Intra	IN	OUT
**CD3+**												
Intra	1	**0.80**	0.47	**0.76**	0.59	0.18	**0.79**	0.64	0.35	0.43	0.41	0.40
IN		1	**0.70**	0.58	0.67	0.28	0.60	**0.75**	0.44	0.42	0.53	0.51
OUT			1	0.19	0.34	0.32	0.27	0.47	0.54	0.23	0.31	0.37
**CD4+**												
Intra				1	**0.80**	0.49	0.69	0.49	0.23	0.43	0.47	0.44
IN					1	**0.67**	0.49	0.63	0.36	0.41	0.55	0.46
OUT						1	0.25	0.37	0.49	0.20	0.34	0.35
**CD8+**												
Intra							1	**0.72**	0.52	0.27	0.22	0.27
IN								1	0.68	0.33	0.44	0.46
OUT									1	0.19	0.25	0.32
**CD68+**												
Intra										1	**0.83**	**0.78**
IN											1	**0.88**
OUT												1

Table S5. Associations between biomarkers and clinical characteristics in the validation cohort. Chi-square and Wilcoxon test.

	Stage at diagnosis – n (%)			Breslow thickness (mm)		Ulceration – n (%)		
	**Stage I/II**	**Stage III/IV**	**P-value**	**Median (Q1-Q3)**	**P-value**	**Absent**	**Present**	**P-value**
**Intratumoral**			0.326 [a]		0.002*			0.414
**Low CD3+**	33 (52.4)	8 (72.7)		4.9 (3.5-8.0)		9 (47.4)	32 (58.2)	
**High CD3+**	30 (47.6)	3 (27.3)		3.3 (2.6-4.0)		10 (52.6)	23 (41.8)	
**Peritumoral IN**			1.000[a]		0.018*			0.929
**Low CD3+**	41 (67.2)	7 (70.0)		4.4 (3.2 -6.0)		13 (68.4)	35 (67.3)	
**High CD3+**	20 (32.8)	3 (30.0)		3.2 (2.6-4.3)		6 (31.6)	17 (32.7)	
**Missing**	2	1				0	3	
**Peritumoral OUT**			1.000 [a]		0.341			1.000 [a]
**Low CD3+**	60 (98.4)	10 (100.0)		4.0 (3.0-5.9)		19 (100.0)	51 (98.1)	
**High CD3+**	1 (1.6)	0 (0.0)		2.9 (2.9-2.9)		0 (0.0)	1 (1.9)	
**Missing**	2	1				0	3	
**Intratumoral**			0.340 [a]		0.006*			0.101
**Low CD4+**	35 (55.6)	8 (72.7)		4.7 (3.2-8.0)		8 (42.1)	35 (63.6)	
**High CD4+**	28 (44.4)	3 (27.3)		3.4 (2.4-4.5)		11 (57.9)	20 (36.4)	
**Peritumoral IN**			1.000 [a]		0.020*			0.734
**Low CD4+**	43 (70.5)	7 (77.8)		4.3 (3.2-6.0)		13 (68.4)	37 (72.5)	
**High CD4+**	18 (29.5)	2 (22.2)		3.3 (2.5-4.1)		6 (31.6)	14 (27.5)	
**Missing**	2	2				0	4	
**Peritumoral OUT**			1.000 [a]		0.748			1.000 [a]
**Low CD4+**	60 (98.4)	9 (100.0)		4.0 (3.0-5.7)		19 (100.0)	50 (98.0)	
**High CD4+**	1 (1.6)	0 (0.0)		3.4 (3.4-3.4)		0 (0.0)	1 (2.0)	
**Missing**	2	2				0	4	
**Intratumoral**			1.000 [a]		0.013*			0.397
**Low CD8+**	38 (60.3)	7 (63.6)		4.6 (3.2-7.3)		10 (52.6)	35 (63.6)	
**High CD8+**	25 (39.7)	4 (36.4)		3.3 (2.6-4.5)		9 (47.4)	20 (36.4)	
**Peritumoral IN**			0.724 [a]		0.001*			0.371
**Low CD8+**	41 (67.2)	6 (60.0)		4.6 (3.4-7.3)		11 (57.9)	36 (69.2)	
**High CD8+**	20 (32.8)	4 (40.0)		3.1 (2.4-3.9)		8 (42.1)	16 (30.8)	

	Stage at diagnosis – n (%)			Breslow thickness (mm)		Ulceration – n (%)		
	**Stage I/II**	**Stage III/IV**	**P-value**	**Median (Q1-Q3)**	**P-value**	**Absent**	**Present**	**P-value**
**Missing**	2	1				0	3	
**Peritumoral OUT**			1.000 [a]		0.295			0.568 [a]
**Low CD8+**	57 (93.4)	10 (100.0)		4.0 (3.0-6.0)		19 (100.0)	48 (92.3)	
**High CD8+**	4 (6.6)	0 (0.0)		3.2 (2.8-4.0)		0 (0.0)	4 (7.7)	
**Missing**	2	1				0	3	
**Intratumoral**			0.752 [a]		0.440			0.291
**Low CD68+**	27 (42.8)	4 (36.4)		4.2 (3.2-6.0)		6 (31.6)	25 (45.5)	
**High CD68+**	36 (57.1)	7 (63.6)		3.9 (2.9-5.7)		13 (68.4)	30 (54.5)	
**Peritumoral IN**			1.000 [a]		0.149			0.981
**Low CD68+**	22 (36.1)	4 (40.0)		4.5 (3.2-6.0)		7 (36.8)	19 (36.5)	
**High CD68+**	39 (63.9)	6 (60.0)		3.5 (2.9-5.3)		12 (63.2)	33 (63.5)	
**Missing**	2	1				0	3	
**Peritumoral OUT**			0.093 [a]		0.501			1.000 [a]
**Low CD68+**	59 (96.7)	8 (80.0)		4.0 (3.0-5.9)		18 (94.7)	49 (94.2)	
**High CD68+**	2 (3.3)	2 (20.0)		3.1 (2.5-7.8)		1 (5.3)	3 (5.8)	
**Missing**	2	1				0	3	

[a]: Fisher exact test. * Significant p-value at 0.05 level.

Table S6. Effect of biomarkers density on disease free survival and overall survival in the validation cohort. Univariable and multivariable Cox proportional hazard models.

	DISEASE FREE SURVIVAL				OVERALL SURVIVAL			
	Univariable analysis		Multivariable analysis		Univariable analysis		Multivariable analysis	
	HR (95% CI)	P-value	HR (95% CI)	P-value	HR (95% CI)	P-value	HR (95% CI)	P-value
**CD3+**								
**Intratumoral** *(100 cell/mm2 increase)*	**0.93 (0.89-0.98)**	**0.005***	**0.95 (0.91-0.99)**	**0.027***	**0.94 (0.89-0.98)**	**0.011***	0.96 (0.91-1.01)	0.113
**Intratumoral** *(high vs low)*	**0.31 (0.16-0.63)**	**0.001***	**0.38 (0.19-0.78)**	**0.008***	**0.36 (0.17-0.75)**	**0.006***	0.53 (0.25-1.13)	0.101
**Peritumoral IN** *(100 cell/mm2 increase)*	**0.96 (0.93-0.99)**	**0.005***	**0.97 (0.93-1.00)**	**0.030***	**0.96 (0.92-0.99)**	**0.011***	0.97 (0.94-1.00)	0.071
**Peritumoral IN** *(high vs low)*	0.67 (0.33-1.38)	0.282	0.73 (0.35-1.51)	0.396	0.68 (0.32-1.47)	0.331	0.77 (0.36-1.68)	0.516
**Peritumoral OUT** *(100 cell/mm2 increase)*	0.90 (0.79-1.02)	0.090	0.89 (0.77-1.02)	0.094	0.92 (0.81-1.04)	0.198	0.91 (0.79-1.04)	0.177
**Peritumoral OUT** *(high vs low)*	Not estimable (see Table 2)		Not estimable (see Table 2)		Not estimable (see Table 2)		Not estimable (see Table 2)	
**CD4+**								
**Intratumoral** *(100 cell/mm2 increase)*	0.99 (0.96-1.01)	0.267	1.00 (0.97-1.02)	0.711	0.99 (0.97-1.02)	0.546	1.01 (0.98-1.03)	0.624
**Intratumoral** *(high vs low)*	0.73 (0.38-1.38)	0.332	1.14 (0.56-2.31)	0.723	0.84 (0.43-1.64)	0.607	1.43 (0.69-2.97)	0.342
**Peritumoral IN** *(100 cell/mm2 increase)*	0.98 (0.95-1.01)	0.129	0.99 (0.96-1.02)	0.392	0.98 (0.96-1.01)	0.302	1.00 (0.97-1.03)	0.860
**Peritumoral IN** *(high vs low)*	0.58 (0.26-1.26)	0.169	0.73 (0.32-1.65)	0.450	0.63 (0.27-1.45)	0.279	0.90 (0.37-2.18)	0.815
**Peritumoral OUT** *(100 cell/mm2 increase)*	1.04 (0.97-1.12)	0.268	1.05 (0.97-1.14)	0.262	1.07 (1.00-1.16)	0.058	1.08 (1.00-1.18)	0.064
**Peritumoral OUT** *(high vs low)*	Not estimable (see Table 2)		Not estimable (see Table 2)		Not estimable (see Table 2)		Not estimable (see Table 2)	
**CD8+**								
**Intratumoral** *(100 cell/mm2 increase)*	**0.92 (0.87-0.98)**	**0.009***	**0.94 (0.88-0.99)**	**0.030***	**0.93 (0.87-0.99)**	**0.026***	0.95 (0.89-1.01)	0.120
**Intratumoral** *(high vs low)*	**0.22 (0.10-0.49)**	**<0.001***	**0.23 (0.10-0.50)**	**<0.001***	**0.25 (0.11-0.57)**	**0.001***	**0.30 (0.13-0.70)**	**0.005***
**Peritumoral IN** *(100 cell/mm2 increase)*	**0.92 (0.88-0.97)**	**0.003***	**0.93 (0.88-0.98)**	**0.006***	**0.93 (0.88-0.98)**	**0.006***	**0.93 (0.88-0.99)**	**0.015***
**Peritumoral IN** *(high vs low)*	**0.22 (0.09-0.53)**	**<0.001***	**0.21 (0.08-0.52)**	**<0.001***	**0.22 (0.08-0.57)**	**0.002***	**0.23 (0.08-0.61)**	**0.003***
**Peritumoral OUT** *(100 cell/mm2 increase)*	0.82 (0.66-1.02)	0.073	0.78 (0.61-1.00)	0.052	0.84 (0.68-1.04)	0.118	0.80 (0.62-1.03)	0.078

	DISEASE FREE SURVIVAL				OVERALL SURVIVAL			
	Univariable analysis		Multivariable analysis		Univariable analysis		Multivariable analysis	
	**HR (95% CI)**	**P-value**	**HR (95% CI)**	**P-value**	**HR (95% CI)**	**P-value**	**HR (95% CI)**	**P-value**
**Peritumoral OUT** *(high vs low)*	Not estimable (see Table 2)		Not estimable (see Table 2)		Not estimable (see Table 2)		Not estimable (see Table 2)	
**CD68+**								
**Intratumoral** *(100 cell/mm2 increase)*	1.00 (0.99-1.00)	0.773	1.00 (0.99-1.00)	0.629	1.00 (0.99-1.01)	0.977	1.00 (0.99-1.01)	0.819
**Intratumoral** *(high vs low)*	0.73 (0.39-1.37)	0.329	0.68 (0.36-1.29)	0.238	0.78 (0.41-1.50)	0.461	0.86 (0.44-1.67)	0.658
**Peritumoral IN** *(100 cell/mm2 increase)*	1.00 (0.99-1.00)	0.475	1.00 (0.99-1.00)	0.643	1.00 (0.99-1.00)	0.762	1.00 (1.00-1.01)	0.778
**Peritumoral IN** *(high vs low)*	0.87 (0.45-1.67)	0.672	0.99 (0.51-1.94)	0.984	0.94 (0.47-1.88)	0.861	1.13 (0.56-2.28)	0.735
**Peritumoral OUT** *(100 cell/mm2 increase)*	1.00 (0.97-1.03)	0.864	0.99 (0.96-1.02)	0.452	1.00 (0.97-1.03)	0.935	1.00 (0.97-1.02)	0.781
**Peritumoral OUT** *(high vs low)*	0.88 (0.21-3.67)	0.864	0.54 (0.12-2.37)	0.417	0.93 (0.22-3.88)	0.919	0.61 (0.14-2.67)	0.512

Note. Multivariable models adjusted for Breslow thickness, ulceration and stage. * Significant p-value at 0.05 level.
